# Robotic-assisted sigmoidectomy with intracorporeal anastomosis and endoscopic management of deep infiltrating intestinal endometriosis

**DOI:** 10.1093/jscr/rjad342

**Published:** 2023-06-10

**Authors:** David Lomeli-Reyes, Jesús Montoya-Ramírez, Enrique Reyes-Rodríguez, Perla X López-Almanza, Paola L Ochoa-Ruiz

**Affiliations:** General Surgery Department, Medica Sur Hospital14050 Mexico City, Mexico; General Surgery Department, Medica Sur Hospital14050 Mexico City, Mexico; General Surgery Department, Medica Sur Hospital14050 Mexico City, Mexico; General Surgery Department, Medica Sur Hospital14050 Mexico City, Mexico; General Surgery Department, Medica Sur Hospital14050 Mexico City, Mexico

## Abstract

Deep endometriosis, defined as external adenomyosis, is a late stage of endometriosis. Associated with very severe pain, in addition to probably being a cofactor of infertility, with a low prevalence, the diagnosis is integrated by high clinical suspicion and confirmed with imaging studies. Deep infiltration can reach sigmoid colon, which would have surgical indication as resolving treatment. We report the case of deep infiltrating endometriosis affecting sigmoid colon of a 42-year-old woman, who was diagnosed with colicky pain in the left lower quadrant and chronic constipation. Colonoscopy revealed a 90% stenosis in the proximal portion of sigmoid colon, as well as mural thickening proximal to the site of stenosis, reported by computed tomography with oral contrast, for which it was decided to perform robot-assisted sigmoidectomy, with a 6-month follow-up and with imaging control, patient continues asymptomatic and without the presence of lesions suggestive of recurrence, and there is no functional impairment.

## INTRODUCTION

Endometriosis is a very common disease in women, defined by the presence of endometrial tissue outside the uterine cavity, a process that induces a chronic inflammatory reaction in tissues and organs [1]. It is estimated that up to 15% of reproductive aged women are diagnosed with endometriosis [2]. In 3–37% of cases, the disease involves the gastrointestinal tract, specifically the bowel and in 90% of these cases, the rectum or sigmoid colon [3, 4]. Intestinal endometriosis is classified as superficial or deep, the latter being the most complex and clinically challenging given it is usually associated with fibrosis and intra-abdominal adhesions. Deep endometriosis specifically, is when the ectopic endometrial tissue is located more than 5 mm below the peritoneal surface. It is most found in the sacroiliac joint ligaments, rectovaginal septum, peritoneum, ureters and bladder [5]. The most frequent anatomic site of deep intestinal endometriosis implantation is the distal portion of the large bowel, specifically the rectum and the rectosigmoid junction in 65.7% of cases, followed by the sigmoid colon in 17.4% of cases [5, 6].

## CASE REPORT

**Figure 1:**
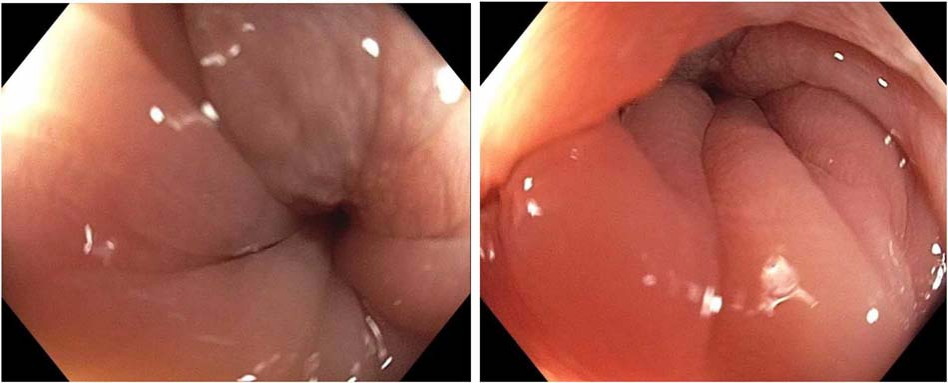
Preoperative colonoscopy: 90% stenosis conditioned to sigmoid colon.

**Figure 2:**
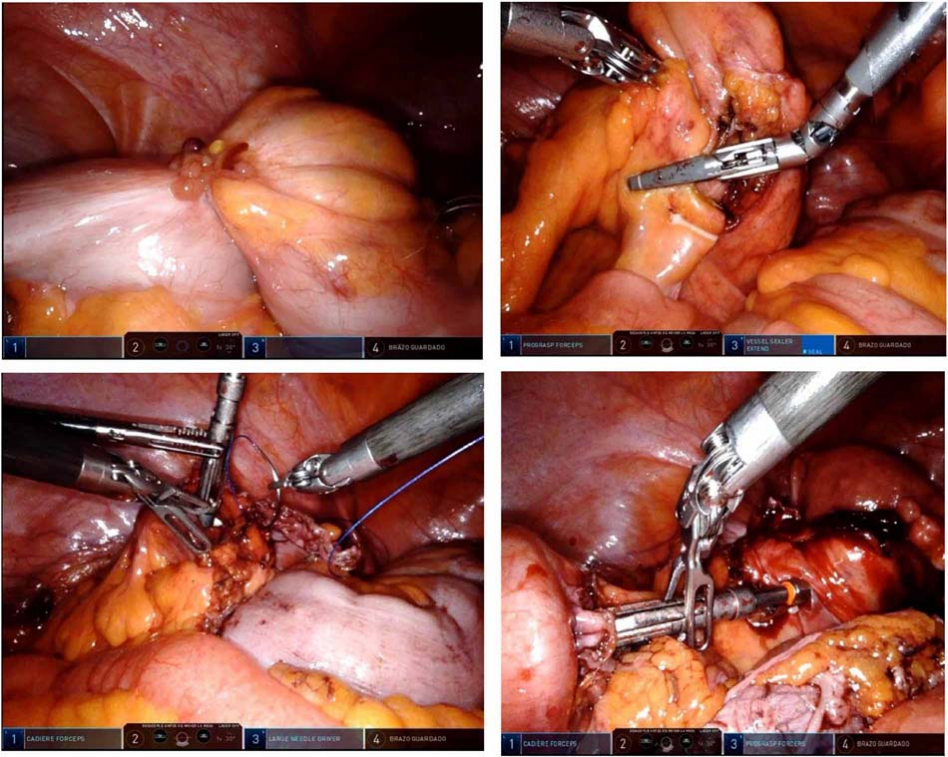
Transoperative findings.

**Figure 3:**
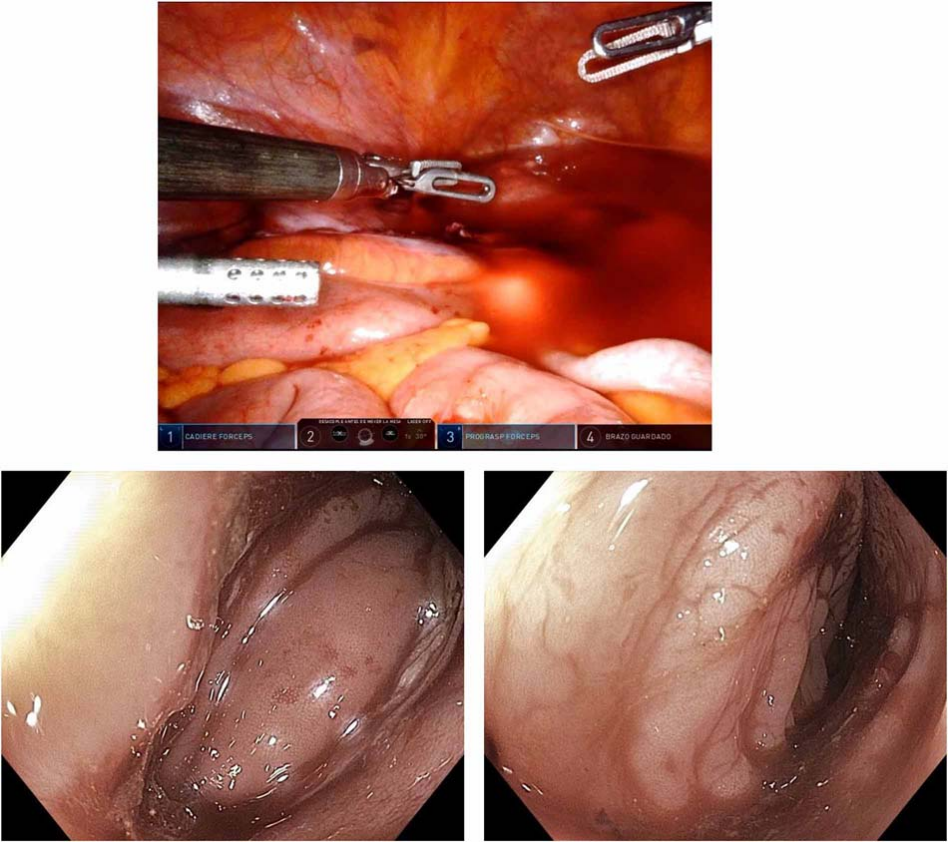
Transoperative colonoscopy.

**Figure 4:**
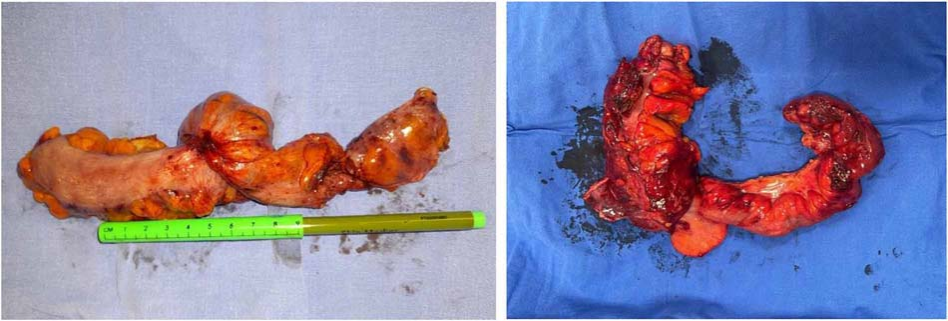
Surgical piece.

**Figure 5:**
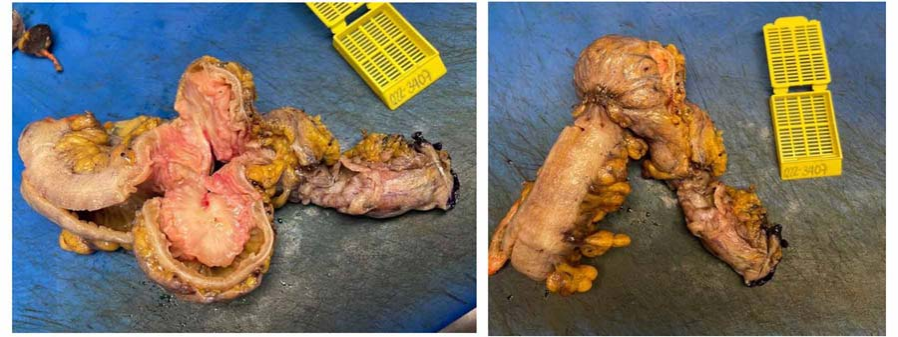
Macroscopic findings.

**Figure 6:**
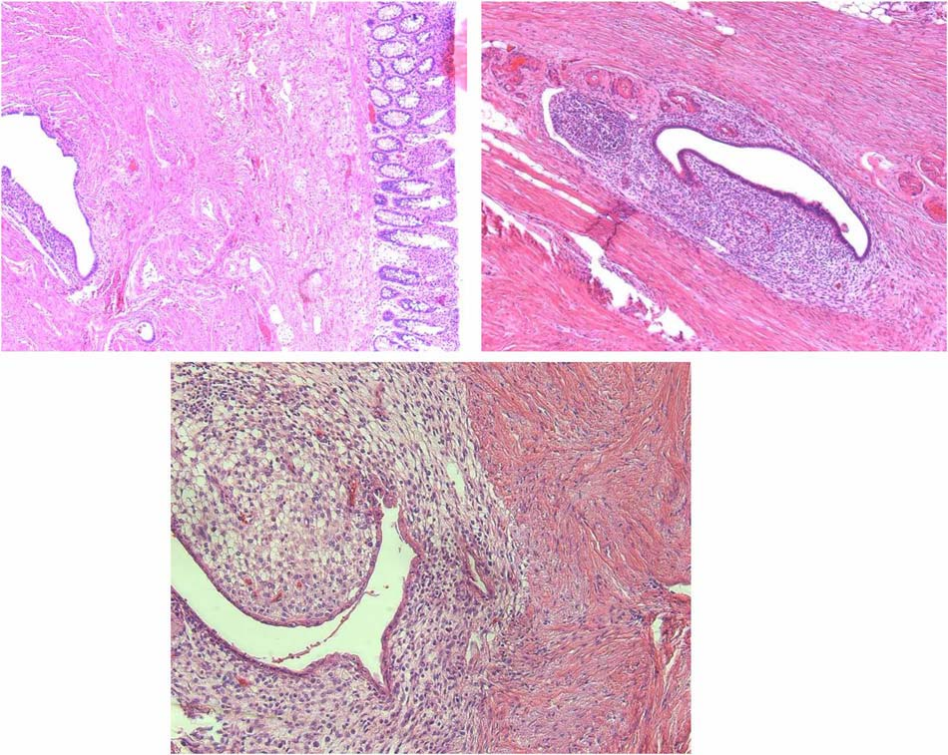
Microscopic histopathological findings.

We present the case of a 42-year-old woman who was diagnosed with chronic constipation that had lasted 5 years. Her chief complaint was absence of stool, intense colicky abdominal pain localised at the left lower quadrant and hypogastrium, nausea and vomiting on five occasions. She consulted her primary physician, who prescribed oral trimebutine, rifaximin, omeprazole and pinaverium bromide with lysine clonixinate; this partially resolved the symptoms. Upon arrival to our medical unit, we conducted laboratory and imaging studies which yielded the following relevant results: leukocytosis (11 400 leukocytes) with neutrophilia (8500 neutrophils), hyperbilirubinemia, (total bilirubin 1.71 mg/dL, direct bilirubin 0.58 mg/dL). The rest of the blood laboratory findings were within normal limits. The urinalysis reported a density of 1015, pH 5.5, ketones +, bilirubin 2 +, positive nitrites, leukocytes of 6–10 and erythrocytes 0–2 per field. Due to persistence of the patient’s symptoms, 2 days later, an abdominal computed tomography (CT) scan with oral contrast was performed. The findings were of a thickened sigmoid colon with associated stenosis. After 3 days, we performed a colonoscopy with sigmoid biopsy (Fig. 1). The results of the histopathological analysis were: chronic nonspecific colitis, edema of the lamina propria and sigmoid stenosis of 90%. The patient was scheduled for a sigmoidectomy with colorectal anastomosis, performed under robotic-assisted surgery. The intraoperative findings were as follows: a sigmoid colon tumor (Fig. 2) of approximately 4 cm × 3 cm with intraluminal infiltration and associated intestinal torsion. The colorectoanastomosis was made 25 cm from the anal margin with no leaks corroborated by an intraoperative colonoscopy (Fig. 3). The definitive pathology findings (Fig. 4) reported a 5 cm × 4.5 cm deep infiltrating intestinal endometriosis (Fig. 5) sigmoid tumor, associated with moderate degree chronic colitis (Fig. 6).

The patient completed the postoperative period with adequate evolution, and hospital discharge 5 days later with no complications.

## DISCUSSION

Deep intestinal endometriosis presents as a clinical picture similar or imitating a colorectal neoplasia; given this similarity in the clinical presentation, physicians must keep in mind the possibility of deep intestinal endometriosis as a differential diagnosis in women diagnosed with constipation, rectal bleeding, changes in bowel habits and intestinal obstruction. In a study conducted by Rossini *et al.*, it was found that there is no significant correlation between symptoms and degree of intestinal wall infiltration [7]. In other words, a highly infiltrative lesion can cause a clinically indolent picture that could only be diagnosed when most of the intestinal lumen is compromised. Regarding the diagnosis of deep intestinal endometriosis, a transvaginal ultrasound has been shown to be effective in detecting intestinal implantation of endometrial tissue, which would be seen as a hypoechoic heterogeneous mass compressing the intestinal lumen [8]. The established sensitivity of this diagnostic test has been reported to be approximately > 85% [9]. Depending on the level of infiltration to the intestinal wall, there are two different surgical approaches. The most conservative technique is nodule resection, whereas laparoscopic intestinal segmental resection is the more radical approach [10]. This decision should not be made preoperatively, but rather transoperative [11]. The degree of infiltration and an extension to > 50% of the circumference have been suggested as clear indications for preferring an intestinal resection [12]. CT scanner and magnetic resonance imaging is recommended in the preoperative workup of deep endometriosis, especially for gastrointestinal endometriosis [13, 14].

According to several studies, laparoscopic rectal and sigmoid resection for deep endometriosis is safe, with low rates of severe complications and a short recovery time [11, 14]. Robotic-assisted laparoscopic surgery has opened up new perspectives for the treatment of endometriosis, as it has been shown to offer a superior advantage, particularly in diffuse extragenital endometriosis [15]. Deep intestinal endometriosis in a female patient with data on intestinal obstruction, changes in bowel habit and decrease in hemoglobin secondary to bleeding from the lower digestive tract. The surgical treatment of deep endometriosis affecting the sigmoid colon must be individualised, seeking intestinal preservation in cases where open, laparoscopic and/or robotic-assisted resection of endometriotic implants is possible, and opting for a resection intestinal in those cases that extensively compromise intestinal viability.
